# Metformin Induces Cell Cycle Arrest and Apoptosis in Drug-Resistant Leukemia Cells

**DOI:** 10.1155/2015/516460

**Published:** 2015-11-25

**Authors:** A. Rodríguez-Lirio, G. Pérez-Yarza, M. R. Fernández-Suárez, E. Alonso-Tejerina, M. D. Boyano, A. Asumendi

**Affiliations:** Department of Cell Biology and Histology, School of Medicine and Dentistry, University of the Basque Country, Leioa, 48940 Bizkaia, Spain

## Abstract

Recent epidemiological studies indicate that the antidiabetic drug metformin has chemosensitizing and chemopreventive effects against carcinogenesis. Here, we demonstrate that metformin exerts varying degrees of antitumor activity against human leukemia cells, as reflected by differences in growth inhibition, apoptosis, and alterations to metabolic enzymes. In metformin-sensitive cells, autophagy was not induced but rather it blocked proliferation by means of arresting cells in the S and G2/M phases which was associated with the downregulation of cyclin A, cyclin B1, and cdc2, but not that of cyclin E. In 10E_1_-CEM cells that overexpress Bcl-2 and are drug-resistant, the effect of metformin on proliferation was more pronounced, also inducing the activation of the caspases 3/7 and hence apoptosis. In all sensitive cells, metformin decreased the Δ*ψ*_*m*_ and it modified the expression of enzymes involved in energy metabolism: PKC*ε* (PKCepsilon) and PKC*δ* (PKCdelta). In sensitive cells, metformin altered PKC*ε* and PKC*δ* expression leading to a predominance of PKC*ε* over PKC*δ* which implies a more glycolytic state. The opposite occurs in the nonresponsive cells. In conclusion, we provide new insights into the activity of metformin as an antitumoral agent in leukemia cells that could be related to its capability to modulate energy metabolism.

## 1. Introduction

Acute lymphoblastic leukemia (ALL) types are aggressive hematological cancers, characterized by the uncontrolled clonal proliferation of immature lymphoid cells at different stages of differentiation and their infiltration of the bone marrow [1]. Approximately 15% of pediatric and 25% of adult ALL cases are of T-cell origin (T-ALL) [2], although adults diagnosed with T-ALL have a worse prognosis than pediatric patients. This difference has been attributed to the development of higher risk leukemia with greater drug resistance and hence a worse response to therapy [3, 4]. Resistance to chemotherapy is an important problem in cancer, representing the main reason for therapeutic failure. Indeed, chemoresistance, either intrinsic or acquired, is believed to cause treatment failure in over 90% of patients with metastatic cancer [5]. Acquired resistance is a particular problem, as tumors not only become resistant to the drugs originally used to treat them but also may become cross-resistant to other drugs with different mechanisms of action. The resistant phenotype represents an adaptive response of cancer cells and it is characterized by alterations to multiple pathways, among which metabolic alterations might play an important role [6]. In T-ALL, Bcl-2 overexpression or mutations in the PTEN protein are related to resistance [7–11].

Taking into account that different metabolic pathways are deregulated in cancer cells, intermediates of these pathways might be excellent candidates for molecular targeting [12–15]. Proliferating cells have distinct metabolic requirements to most normal differentiated cells [13] and thus many key oncogenic signaling pathways converge and modify tumor cell metabolism in order to support their growth and survival [14]. Tumor cells preferentially use glycolysis over mitochondrial oxidative phosphorylation for glucose-dependent ATP production, even in the presence of oxygen to fuel mitochondrial respiration (Warburg effect) [12]. Moreover, tumors exhibit heterogeneous metabolic alterations that extend beyond the Warburg effect [14], which may represent an opportunity for novel therapies [16]. In this sense, antitumoral therapies targeting cell metabolism have been investigated, such as the use of biguanides.

Metformin (1,1-dimethylbiguanide) belongs to the biguanide class of oral hypoglycemic agents that has been used widely for many years in the treatment of type 2 diabetes [17]. Intriguingly, there is a growing body of evidence that metformin also has chemosensitizing and chemopreventive effects against carcinogenesis in general [18–21]. The antitumoral effects of metformin are associated with both direct (insulin-independent) and indirect (insulin-dependent) actions of the drug. The insulin-dependent effects of metformin are based on its ability to inhibit hepatic gluconeogenesis and to stimulate glucose uptake in muscle and adipocytes, thereby lowering the glucose and insulin levels in the blood. This effect of metformin on insulin is important in the treatment of hyperinsulinemia-related tumors (insulin-responsive tumors) [22]. Metformin also inhibits mitochondrial oxidative phosphorylation due to the disruption of respiratory complex I, provoking energetic stress due to reduced ATP production in the mitochondria and the ensuing activation of the LKB1/AMPK pathway [23]. AMPK acts as a metabolic sensor, controlling cell metabolism and growth, autophagy, and cell polarity in conditions of low energy [24, 25]. Importantly, AMPK inhibits mTOR through distinct mechanisms, dampening the phosphorylation of its downstream effectors 4E-BP and S6K, and inhibiting protein synthesis and proliferation [22, 24, 25]. Moreover, activated AMPK stimulates catabolic processes that generate ATP (glycolysis and fatty acid *β*-oxidation) and that inhibit anabolic process which consume ATP to restore a normal ATP/AMP ratio (gluconeogenesis, protein and fatty acid synthesis, and cholesterol biosynthesis) [22].

The protein kinase C (PKC) family of serine/threonine kinases plays critical roles in the transduction of signals that affect cell proliferation, survival, differentiation, and apoptosis, and these kinases are attractive therapeutic targets in many cancers. Due to their distinct subcellular localization and tissue distribution, each PKC displays particular signaling characteristics [26]. PKC*δ* has emerged as a novel regulator of oxidative phosphorylation that targets the pyruvate dehydrogenase complex (PDHC). PKC*δ* activation leads to pyruvate dehydrogenase kinase 2 (PDK2) dephosphorylation, and this decrease in PDK2 activity and the ensuing increase in PDHC activity accelerate oxygen consumption and augment ATP synthesis [27]. A second PKC isoform, PKC*ε*, is thought to oppose the action of PKC*δ* in mitochondria [28]. Moreover, PDHC was recently identified as a common downstream target that was stimulated by PKC*δ* or inhibited by PKC*ε* signaling [29]. The PDHC is the mitochondrial entry point of the glycolytic end product pyruvate and thus it is of crucial importance to fuel metabolic energy flow.

In this study we assessed the antitumor activity of metformin on human ALL cells that are resistant to other antineoplastic drugs, analyzing its capacity to inhibit growth, apoptosis, or autophagy. Moreover, in the light of evidence regarding metformin's influence on metabolism, we also explored the alterations provoked by metformin to energy metabolism. Our results suggest that the efficacy of metformin against ALL could be related to its ability to disturb the balance between PKC*ε* and PKC*δ*, two important kinases recently proposed to be crucial for energy homeostasis.

## 2. Materials and Methods

### 2.1. Chemicals

RPMI medium 1640 (1x) + GlutaMAX was obtained from Gibco (Live Technologies) and Fetal Bovine Serum (FBS) from Biochrom AG (Berlin, Germany), and propidium iodide (PI), 4′-6-diamidino-2-phenylindole (DAPI), bicinchoninic acid solution, and gentamicin were obtained from Sigma-Aldrich Quimica, S.A. (Madrid, Spain). Metformin and the annexin V-FITC Apoptosis Detection Kit were purchased from Calbiochem (Darmstadt, Germany) and the XTT Cell Proliferation Kit II and RNase from Roche Molecular Biochemicals (Indianapolis, IN, USA). The Vybrant FAM caspase-3 and caspase-7 assay kit and DiOC_6_(3) were obtained from Molecular Probes (Live Technologies).

The rabbit polyclonal anti-cyclin A (1 : 500, sc-751), anti-cyclin E (1 : 500, sc-481), anti-PKC*ε* (1 : 200, sc-214), and anti-PKC*δ* (1 : 200, sc-213) antibodies and the mouse monoclonal anti-cyclin B1 (1 : 500, sc-245) and anti-cdc2 p34 (1 : 500, sc-54) antibodies were obtained from Santa Cruz Biotechnology (Santa Cruz, CA, USA). The rabbit polyclonal anti-LC3B (1 : 3000, ab51520) and the HRP-conjugated goat secondary polyclonal antibody against rabbit IgG-H&L (1 : 3000, ab6721) were purchased from Abcam (Cambridge, UK). Goat F(ab′)_2_ anti-mouse IgG (H+L) was obtained from Southern Biotech (1032-05) and the rabbit anti-actin (1 : 200, A2066) antibody was from Sigma-Aldrich Quimica, S.A. (Madrid, Spain).

### 2.2. Cell Lines and Culture Conditions

Human ALL CEM cells were purchased from ATCC. 10E_1_-CEM cells are CEM cells transfected with a vector containing a Bcl-2 cDNA (kindly provided by Dr. Kofler) [30]. We previously demonstrated that 10E_1_-CEM cells were resistant to 4-HPR (4-hydroxy(phenyl)retinamide or fenretinide) treatment [31]. Both of these cell lines were cultured in RPMI medium 1640 (1x) + GlutaMAX, supplemented with 10% (v/v) heat inactivated FBS and 100 *μ*g/mL gentamicin, and the cells were maintained at 37°C in a humidified incubator containing 5% CO_2_. R5-CEM are 4-HPR-resistant cells derived from the parental CEM cells by exposure to increasing concentrations of 4-HPR in culture, as described elsewhere [32]. Cells in the exponential growth phase were used in all experiments.

### 2.3. Cell Viability Assay

A standard XTT assay was used to evaluate cell viability. Cells were seeded in 96-well plates at a density of 500,000 cells/mL in complete culture medium, 100 *μ*L per well, and 50 *μ*L of different final concentrations of metformin in complete medium was then added. After various times in culture, the XTT reagent mixture was added and absorbance at 490 nm was determined 4 hours later in a microplate reader (Synergy HT, BioTek, Germany), according to the manufacturer's instructions. Four replicates were used per experimental condition and the percentage of cell viability was determined in reference to control untreated cells.

### 2.4. Cell Cycle Analysis

The effect of metformin on cell cycle progression was evaluated by DNA flow cytometry of PI stained cells. Cells were cultured in 6-well plates and treated with the metformin concentrations indicated. Cells were collected at different times after the treatment, washed in PBS, and fixed with cold 70% ethanol overnight at −20°C. The cells were then washed with PBS and treated with 50 *μ*g/mL PI and 200 *μ*g/mL RNase A for 30 min at 37°C. Cell cycle analysis was performed on a Coulter EPICS ELITE ESP flow cytometer (EPICS Division Coulter Corp.) and the results were analyzed using Summit v4.3 software.

### 2.5. Apoptosis Detection by Flow Cytometry

Apoptotic cell death was measured by flow cytometry (EPICS Division Coulter Corp.) using the annexin V-FITC/PI double staining kit (Calbiochem, Germany), according to the manufacturer's instructions. The numbers of viable (annexin negative/PI negative), early apoptotic (annexin positive/PI negative), and late apoptotic/necrotic (annexin and PI positive) cells were determined using Summit v4.3 software, normalized to the basal apoptosis determined on untreated cells. Apoptosis was also studied using the FLICA reagent provided in the caspase-3 and caspase-7 assay kit (Molecular Probes), which evaluates caspase activation in combination with PI staining.

### 2.6. Apoptotic Detection by Nuclear Staining

Changes to nuclear chromatin were evaluated by DAPI staining of treated and untreated cells plated on poly-L-lysine (0.01%) pretreated sterile glass coverslips in 24-well plates. The cells were washed twice with PBS and fixed and permeabilized for 30 min in 70% methanol at −20°C, and after washing with PBS they were incubated for 15 min at room temperature in the dark with DAPI (5 *μ*g/mL) to stain the nuclei. The coverslips were mounted in Fluoromount G mounting medium after labeling, and apoptotic cells were visualized and photographed by fluorescence microscopy (Zeiss Anxioskop run by Nikon NIS-Elements).

### 2.7. Analysis of the Mitochondrial Membrane Potential (Δ*ψ*_*m*_)

Variations of the Δ*ψ*_*m*_ after metformin treatment were evaluated using 3,3′-dihexyloxacarbocyanine iodide fluorochrome (DiOC_6_(3)). After drug exposure, cells were incubated for 20 min at 37°C in the dark with 100 nM DiOC_6_(3) in serum-free RPMI medium. The cells were then washed twice with PBS, exposed to PI (5 *μ*g/mL), and analyzed on a Coulter EPICS ELITE ESP flow cytometer (EPICS Division Coulter Corp.) using Summit v4.3 software. As positive control of Δ*ψ*_*m*_ disturbance, CEM cells treated with 3 *μ*M 4-HPR were used.

### 2.8. Western Blotting

Treated and untreated cells were recovered, washed in cold PBS, and lysed on ice for 15 min with RIPA lysis buffer (150 mM NaCl, 50 mM Tris-HCl [pH 8], 1% Nonidet-P40, 0.5% sodium deoxycholate, and 0.1% sodium dodecyl sulphate) supplemented with protease and phosphatase inhibitors (Sigma-Aldrich Quimica, S.A., Madrid, Spain). The viscosity of the lysates was reduced by shearing the DNA by passing the lysate through a 26 g needle and they were then cleared by centrifugation at 13,000 rpm for 5 min at 4°C. The protein content of the supernatants was determined with the bicinchoninic acid protein assay using bovine serum albumin (BSA) as a standard. Cleared protein lysates from each experimental condition (40 *μ*g) were denatured by boiling for 5 min and resolved by 12% SDS-PAGE. The proteins were transferred to nitrocellulose membrane (Whatman GmbH, Dassel, Germany) and they were blocked for 1 h at room temperature with 5% (w/v) nonfat dry milk or BSA in TBS containing 0.1% (v/v) Tween-20 (TBS-T). The membranes were then probed with the primary antibodies overnight at 4°C and, after washing the membranes in TBS-T, antibody binding was detected with the appropriate horseradish peroxidase-conjugated secondary antibodies for 2 h at room temperature. The immune complexes were visualized by ECL, according to the manufacturer's instructions (SuperSignal West Pico Chemiluminescent Substrate, Thermo Scientific, Rockford, IL, USA), using actin as a loading control. The protein levels were quantified by densitometry and expressed relative to actin.

### 2.9. Statistical Analysis

All the results were expressed as the mean ± standard deviation of at least three independent experiments (the number of replicates was specific to each experiment). The data were compared to the control condition using Student's *t*-test, with *P* values of ≤0.05 (^*∗*^) or ≤0.01 (^*∗∗*^) considered to be statistically significant or very significant.

## 3. Results

### 3.1. Metformin Reduces Human Leukemia Cell Viability

Our aim was to study the effectiveness of metformin on cells that are resistant to other antineoplastic drugs. For this purpose we employed 10E_1_-CEM (Bcl-2 overexpressing cells) and R5-CEM cells, both derived from the human leukemia CEM native cell line and resistant to antineoplastic drug 4-HPR, among others [30–32].

**Figure 1:**
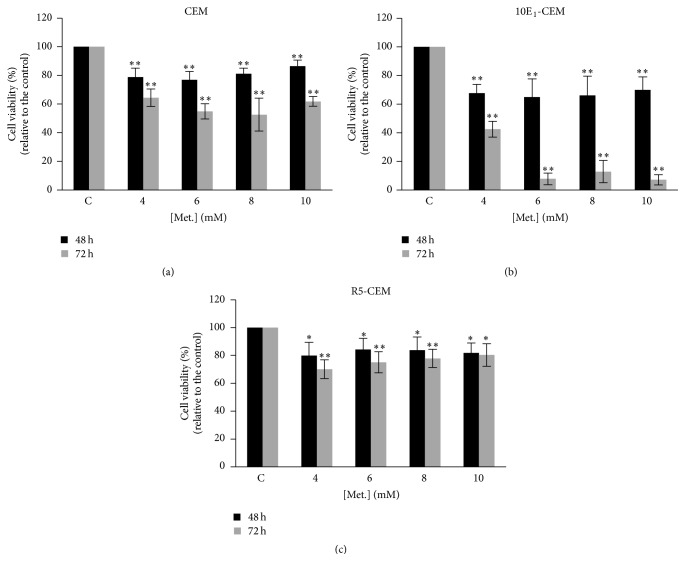
Effect of metformin on leukemia cell viability. The leukemia cell lines CEM, 10E_1_-CEM, and R5-CEM were exposed to metformin for the times and at the concentrations indicated, and cell viability was determined with the XTT assay. The values are shown relative to the untreated control cells (±SD) from at least three independent experiments performed in quadruplicate (*n* > 12): ^*∗∗*^ *P* < 0.01, ^*∗*^ *P* < 0.05 statistically significant differences.

The effect of metformin on the viability of CEM, 10E_1_-CEM, and R5-CEM cells was studied with the XTT assay (Figure 1). We employed mM concentrations of metformin which have very low toxicity and do not affect survival of normal hematopoietic precursors [33, 34]. In all cases, metformin induced a decrease in cell viability compared to untreated cells but in a different extent. The most metformin-sensitive cell lines were the CEM and 10E_1_-CEM, the latter—in which Bcl-2 is overexpressed [30]—being more sensitive to metformin. In both lines, metformin effect was higher at 72 h of treatment. Indeed, while there was a ~40–50% decrease in CEM cell viability after 72 h in the presence of metformin at all concentrations used, the 10E_1_-CEM displayed ~60% decrease in cell viability at the lower doses used, reaching ~90–95% at higher doses. By contrast, only a mild loss of R5-CEM cell viability (~20%) was evident, these cells having acquired resistance to 4-HPR. This is the maximum effect observed in these cells, even at longer times and higher doses of metformin. We have considered R5-CEM cells as nonresponsive to metformin in contrast to more sensitive CEM and 10E_1_-CEM cells.

To determine the mechanism by which metformin impaired cell viability, cell death and proliferation were examined in the most metformin-sensitive cell lines, CEM and 10E_1_-CEM.

### 3.2. Metformin Effect on the Induction of Apoptosis and Autophagy

**Figure 2:**
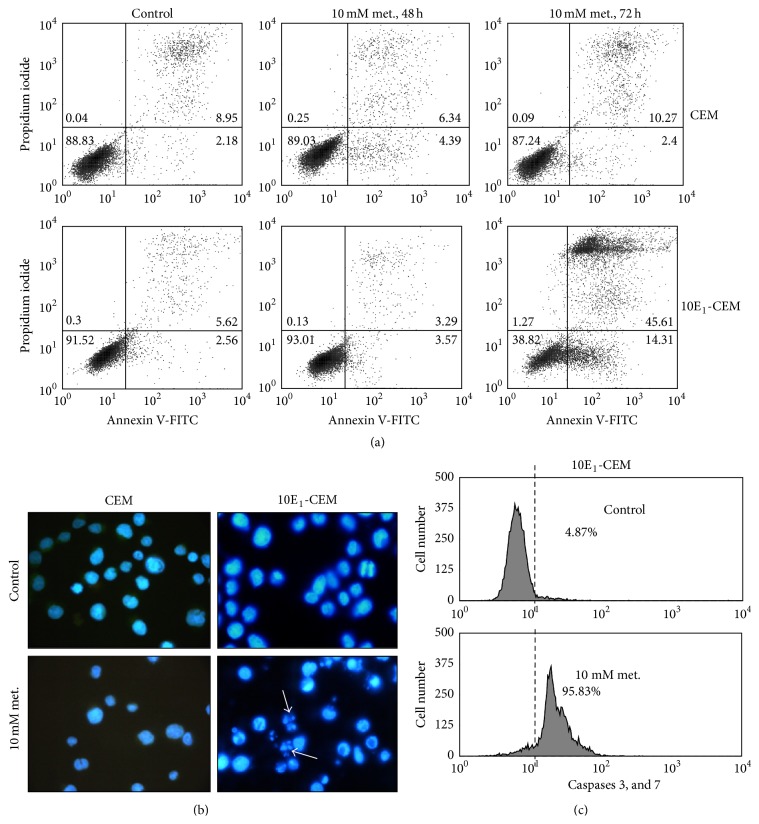
Effects of metformin on apoptosis. CEM and 10E_1_-CEM cells were exposed to 10 mM metformin for 72 h and the induction of apoptosis was evaluated by (a, and Table 1) flow cytometry of annexin V-PI labeled cells and (b) by fluorescence microscopy of DAPI labeled cells. (c) 10E_1_-CEM cells were treated with 10 mM metformin for 96 h before caspase-3 and -7 activation was evaluated by flow cytometry. The numbers in the quadrants indicate the percentages of cells. A representative experiment of three performed. (a, and Table 1).

**Figure 3:**
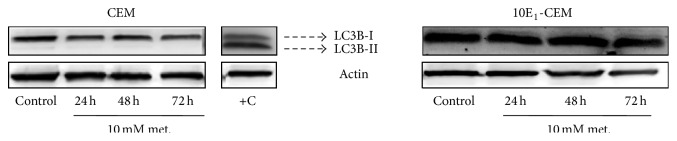
The effect of metformin on the induction of autophagy. Western blots probed for LC3B in CEM and 10E_1_-CEM cells exposed to metformin (10 mM) for the times indicated. Mel-Ho cells treated for 30 h with terfenadine (10 *μ*M) were used as a positive control where conversion of LC3B-I isoform to the LC3B-II is shown.

First we assessed whether the cytotoxicity of metformin involved cell death, apoptosis (Figure 2 and Table 1), and/or autophagy (Figure 3). Flow cytometry analysis of annexin V-PI labeled cells revealed no apoptotic CEM and 10E_1_-CEM cells after 48 h in the presence of metformin (Figure 2(a) and Table 1). By contrast, apoptotic cell death of 10E_1_-CEM cells was evident at 72 h (58.61% of apoptotic cells) but not in CEM cells (12.04% apoptotic cells, not significant). The induction of apoptosis in the most metformin-sensitive 10E_1_-CEM cells was further confirmed by fluorescence microscopy after labeling the cell nuclei with DAPI (Figure 2(b)). Changes in nuclear chromatin were not observed in CEM cells but 10E_1_-CEM cells with an apoptotic morphology were evident, presenting condensed micronuclei (arrows). Moreover, executive caspases 3 and 7 were activated in 95% of these 10E_1_-CEM cells after 96 h in the presence of metformin, as seen by flow cytometry (Figure 2(c)).

To study autophagy, the conversion of the microtubule-associated 1 light-chain 3 (LC3B-I) isoform to the autophagosome-associated LC3B-II was assessed in immunoblots. Exposure to metformin did not induce autophagy in the leukemia cell lines studied (Figure 3), since no LC3B-II was observed in metformin-treated cultures. Hence, autophagy does not appear to contribute to the observed antileukemic activity of metformin, indicating that apoptosis is the sole cell death process induced by metformin.

### 3.3. Metformin Inhibits Cell Proliferation by Arresting Cells in the G2/M and S Phases of the Cell Cycle

**Figure 4:**
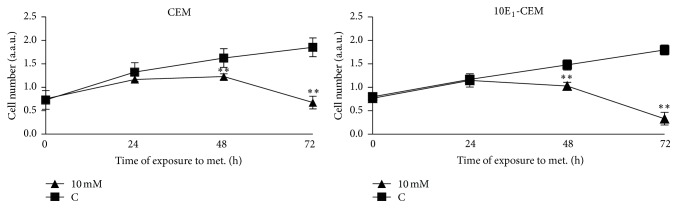
The effect of metformin on leukemia cell proliferation. CEM and 10E_1_-CEM cells were treated with metformin (10 mM) for the times indicated and the viable cell number was determined as an arbitrary unit of absorbance after XTT tetrazolium salt incorporation. Values are represented as the cell number (±SD) relative to the controls of at least three independent experiments performed in quadruplicate (*n* > 12): ^*∗∗*^ *P* < 0.01, ^*∗*^ *P* < 0.05 statistically significant differences.

The effect of metformin on leukemia cell proliferation was studied, demonstrating that metformin significantly inhibited the growth of CEM and 10E_1_-CEM cells in a time-dependent manner (Figure 4). Metformin produced a similar effect on both cell lines during the first 48 hours, provoking ~30% inhibition of cell proliferation, yet this rose to ~80% inhibition of 10E_1_-CEM proliferation at 72 hours. Note that the apoptosis induced in 10E_1_-CEM cells (Figure 2 and Table 1) contributed to this inhibition of proliferation.

**Figure 5:**
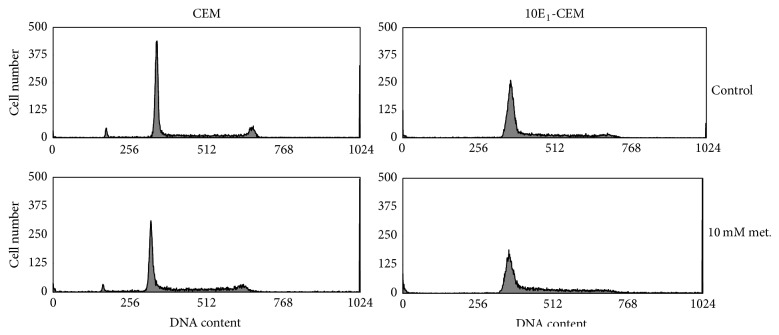
Metformin induces cell cycle arrest. CCRF-CEM and 10E_1_-CEM cells were treated with 4 and 10 mM metformin for 72 h and cell cycle distribution was determined by flow cytometry. Representative histograms showing the distribution of cells on the basis of DNA content.

The distribution of the cells in the different phases of the cell cycle was analyzed by flow cytometry of PI stained cells after a 48 h exposure to metformin. Longer exposures would induce apoptosis of 10E_1_-CEM cells, which could disturb the analysis. Metformin clearly perturbed cell cycle progression, inducing a decrease in the nonproliferative G0/G1 fraction and an increase in the proliferative S and G2/M fraction (Figure 5 and Table 2), reflecting the arrest of proliferative cells. Exposure of CEM cells to metformin for 48 h also led to an accumulation of cells in the S and G2/M phase, rising from ~36% to ~47%, with a corresponding decrease of cells in the G0/G1 phase from ~64% to ~53%. The increase of 10E_1_-CEM cells in the S and G2/M phase on exposure to metformin was more pronounced, rising from ~31% to ~50%, with a corresponding decrease in the G0/G1 phase from ~68% to ~49%. Thus, the inhibition of cell proliferation provoked by a 48 h exposure to metformin is mediated by cell cycle arrest in the G2/M and S phase.

**Figure 6:**
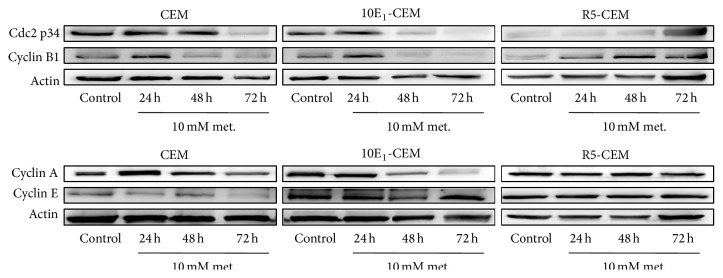
The effect of metformin on cell cycle regulatory proteins. Western blot analysis of cdc2 p34, cyclin A, cyclin B1, and cyclin E in CEM, 10E_1_-CEM, and R5-CEM cells treated with metformin (10 mM) for the times indicated. Representative results of three independent assays performed are shown.

We analyzed the expression of different cell cycle components to confirm these data, including cyclin A, cyclin B1, cyclin E, and cdc2 (Figure 6 and Table 3). Consistent with the observed G2/M and S cell cycle arrest, exposure to metformin diminished cyclin A, cyclin B1, and cdc2 (p34) in both CEM and 10E_1_-CEM cells. The reduction of these cell cycle regulatory proteins was more evident after a 72 h exposure and in 10E_1_-CEM cells, in accordance with the changes in cell proliferation. However, there was no significant reduction in the expression of cyclin E in these cells, suggesting that G0/G1 checkpoint does not contribute to the cell cycle arrest. In R5-CEM cells, as expected, exposure to metformin did not change in the same manner the levels of these regulatory proteins. Quite the opposite, an increment of cyclin A (nonsignificant), B1, and cdc2 (nonsignificant) levels was observed, which is consistent with the mild effect of metformin on such cells. Collectively, these results indicate that metformin inhibits cell cycle progression in CEM and 10E_1_-CEM cells by modulating the expression of cell cycle proteins related to the S and G2/M phases.

### 3.4. Metformin Decreases Δ*ψ*_*m*_ and Induces Changes in Cell Metabolism

**Figure 7:**
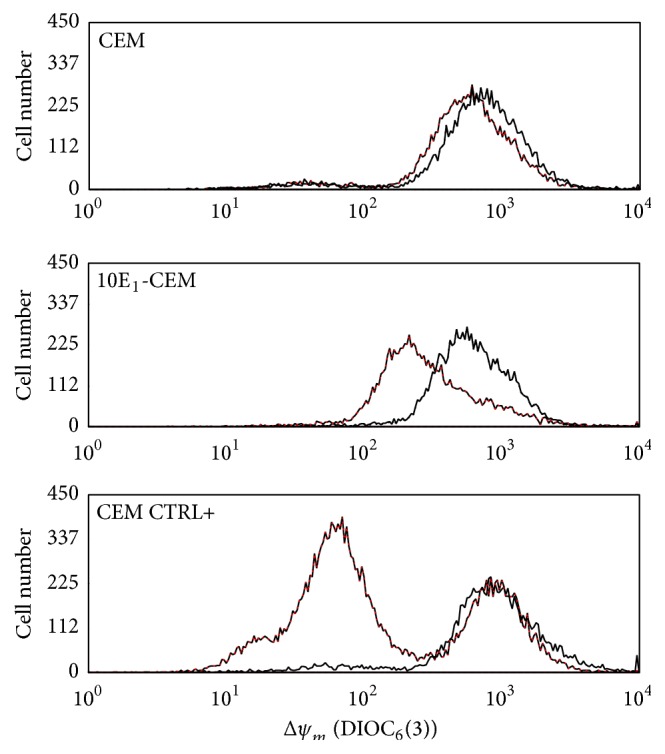
Metformin induces mitochondrial perturbations. CEM and 10E_1_-CEM cells were exposed to metformin (10 mM) for 48 h and then stained with DiOC_6_(3)/PI. The Δ*ψ*_*m*_ was determined as DiOC_3_(6) emitted fluorescence (black: control cells, red: metformin-treated cells). Fluorescence by CEM cells treated with 4-HPR has been shown as positive control (CEM CTRL+). In all cases, differences of mean fluorescence intensity of metformin* versus* control (*n* = 10.000 cells analyzed/assay) are statistically significant (*P* < 0.01) for each experiment of the three performed.

**Figure 8:**
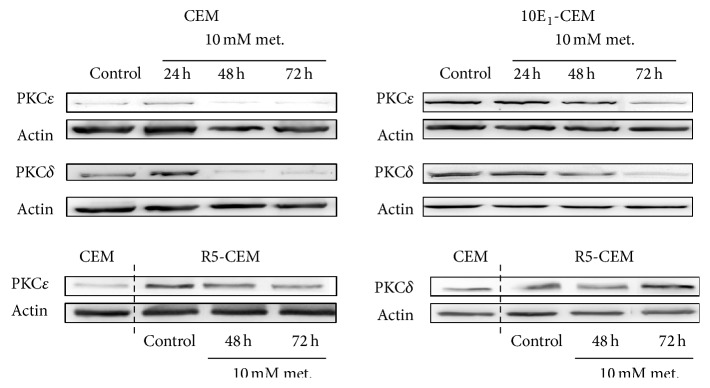
Metformin induces changes in enzymes controlling energy metabolism. Western blots of PKC*δ* and PKC*ε* in CEM, 10E_1_-CEM, and R5-CEM cells exposed to 10 mM metformin for the times indicated. Representative results of three independent assays performed are shown.

Metformin inhibits mitochondrial oxidative phosphorylation by disrupting respiratory complex I [16] and thus we determined Δ*ψ*_*m*_ by assessing the incorporation of DiOC_6_(3) in treated and untreated cells (Figure 7). Metformin induced an early dissipation of Δ*ψ*_*m*_ in CEM and 10E_1_-CEM cells, an effect evident after a 48 h exposure to metformin, more pronounced on 10E_1_-CEM cells. This change reflects the effect of metformin on the mitochondria and the subsequent energetic stress due to reduced ATP production. To further analyze the changes in cell metabolism that may be caused by such energetic stress, we examined the expression of proteins related to energy metabolism. Specifically, we analyzed the PKC*ε* and PKC*δ* proteins that inhibit and stimulate PDHC, respectively (Figure 8 and Table 4).

Metformin induced clear downregulation of PKC*δ* and PKC*ε* in both CEM and 10E_1_-CEM cells (as visualized for one experiment in Figure 8). Both kinases regulate energy homeostasis by transmitting opposite signals to PDHC and the net effect sensed by the PDHC is determined by the relative strength of its two opposing upstream signals [29]. A disturbed balance between PKC*δ* and PKC*ε* favoring the latter implies an increase in glycolytic metabolism over mitochondrial oxidative phosphorylation. This shift toward a more glycolytic metabolism was more pronounced in 10E_1_-CEM cells, in which PKC*δ* expression was most notably reduced in three independent experiments (Table 4). In contrast, in nonresponsive R5-CEM cells, metformin induced downregulation of PKC*ε* and an increase of PKC*δ* expression.

## 4. Discussion

Metformin is the most commonly prescribed antidiabetic drug worldwide [22, 35]. However, several studies in recent years have demonstrated that metformin has antitumoral properties* in vivo* and* in vitro*, and epidemiological evidence suggests it might play an important role in cancer prevention [36, 37]. Accordingly, here we demonstrate the antitumor potential of metformin in human ALL cells, suggesting that it may be effective against leukemia cells that are resistant to other drugs. That is the case of 10E_1_-CEM cells that overexpress Bcl-2 [30] and that are resistant to 4-HPR [31] and BTS (benzo(b)thiophenesulphonamide 1,1-dioxide derivatives) [38], among other drugs. Leukemia types in which Bcl-2 is overexpressed have a poor prognosis and they have traditionally been related to chemoresistance, emphasizing the importance of this finding [7, 39]. Indeed, Bcl-2 overexpression in leukemia types confers resistance to a wide range of antineoplastic drugs [40–44] and this phenomenon has also been observed in other cancers [11]. Metformin has very low toxicity and it does not affect the growth, differentiation, or survival of normal hematopoietic precursors, even at the concentrations up to 120 mM [33, 34]. Thus, it appears to be worthy to study its utility as an antitumoral agent in leukemia in more depth.

Metformin and other biguanides are thought to activate AMPK by acting as mild inhibitors of complex I of the respiratory chain, leading to a drop in intracellular ATP levels [22]. We observed a metformin-induced decrease in Δ*ψ*_*m*_, which probably leads to energetic stress through reduced ATP production. However, sensitivity to metformin differed among the cell lines studied, as reflected in the inhibition of proliferation, apoptosis, and alterations to important enzymes of energy metabolism. These alterations associated with metformin toxicity are more evident in the most sensitive Bcl-2 overexpressing 10E_1_-CEM cells.

While it is accepted that metformin limits the proliferation of cancer cells [21, 45, 46], the molecular mechanisms underlying this effect remain unclear. As shown elsewhere [47–50], we found that metformin induces the arrest of proliferative cells (S-G2/M accumulation) and, indeed, metformin downregulated the expression of cyclin A, cyclin B1, and cdc2 in sensitive cells but not the G0/G1 checkpoint protein cyclin E. G0/G1 cell cycle arrest has been reported to be induced by metformin in glioma, ovarian, and endometrial cancer cells [37, 51, 52], suggesting the specificity of metformin's antiproliferative action [47]. Yet conversely, it does not seem that metformin's action is always linked to apoptosis and autophagy. The proapoptotic activity of metformin in leukemia cells [46, 53] and a lack of apoptotic induction [34, 54] have also been described in sensitive cells. We observed both situations, consistent with the idea that a stronger effect of metformin is related with the induction of apoptosis in addition to growth inhibition. Metformin also targets tumor cells through autophagy, which, while being essential to conserve cell viability in times of metabolic stress, can also promote cell death. We failed to detect activation of autophagy by metformin, even in the most sensitive 10E_1_-CEM cells, while the induction of autophagy was confirmed in ALL cells through AMPK-mTORC1 signaling inhibition [53]. Indeed, it has been reported that metformin only induces autophagy in combination with other chemotherapeutic agents or mTOR inhibitors, yet not alone [34, 54], suggesting that the autophagic response may be defined by the tumors' own metabolic alterations or culture/environmental features (i.e., glucose availability).

Metformin activates the LKB1-AMPK pathway that negatively regulates the mTOR pathway and consequently inhibits the translation of oncogenic proteins [53]. Here we present evidence that the expression of important metabolic kinases other than AMPK is modulated by metformin in ALL cells. Metformin modulates PKC*ε* and *δ* expression in responsive cells resulting in PKC*δ* downregulation and consequently PKC*ε* domination. Both enzymes influence key steps in glucose metabolism and they appear to be uniquely positioned to integrate signaling with metabolism [28]. The balance between PKC*ε* and PKC*δ* may be of paramount importance, not only for the flux entering the Krebs cycle but for overall energy homeostasis [29]. To our knowledge, the effect of metformin on these PKC isoforms has not been described previously, and drugs like metformin that can modulate the PKC*ε* and PKC*δ* balance could have lethal effects on tumor cells with dysregulated energetic metabolism [14]. It was recently revealed that PKC*ε* and PKC*δ* regulate energy homeostasis in mitochondria by transmitting opposing signals to the PDHC [29]. Thus, it is assumed that PKC*ε* predominance leads to a more glycolytic state by inhibiting PDHC. The reduced mitochondrial potential and ATP synthesis induced by metformin could lead to compensatory stimulation of glycolysis to maintain the cell's ATP content normal, the Pasteur effect [55]. In this sense, tumor cells with a deficit in the ability to cope with energetic stress may experience an energetic crisis leading to death [23]. As opposed to sensitive cells, metformin does not downregulate PKC*δ* expression in R5-CEM cells but the opposite effect rather occurs and PKC*ε* downregulation and strong PKC*δ* upregulation were observed leading to PKC*δ* dominance. Interestingly, the basal PKC*ε* in R5-CEM cells is much higher than in the native CEM cells. The PKC*ε* upregulation in R5-CEM cells could be related to the presence of 4-HPR in the medium culture, necessary to maintain the acquired resistance. This is consistent with a recent report showing that chronic use of 4-HPR leads to the predominance of PKC*ε* over PKC*δ* [29, 56].

The alterations to the balance between PKC*ε* and PKC*δ* could also have important consequences for tumor cell survival, since both kinases control the Nrf2 transcription factor [56]. Nrf2 plays an important role in normal cell survival and in drug resistance of cancer cells [56–58]. Constitutive expression of Nrf2 upregulates cytoprotective genes, promotes cell proliferation and chemoresistance, and inhibits apoptosis, potentially conferring a survival advantage to cancer cells [59]. PKC*ε* phosphorylated INrf2 induces Nrf2 degradation [56], while PKC*δ* phosphorylates Nrf2 and drives its translocation to the nucleus, increasing drug resistance [60]. Bearing in mind the control exerted by PKCs in Nrf2, metformin-induced PKC*ε* and PKC*δ* alteration could play an important role in determining the growth inhibition and apoptosis in responsive cells.

## 5. Conclusions

In conclusion, our results demonstrate that metformin has an antitumoral effect on ALL cells, even against drug-resistant cells. Metformin induces cell cycle arrest and apoptosis in drug-resistant leukemia cells.

## Figures and Tables

**Table 1:** Apoptotic cell percentages after metformin treatment.

	Control	10 mM 48 h	10 mM 72 h
CEM			
% viable cells	89.07 ± 1.82	89.39 ± 0.38	86.63 ± 0.43
% apoptotic cells	10.88 ± 1.86	10.38 ± 0.4	12.04 ± 1.7
10E_1_-CEM			
% viable cells	91.49 ± 0.15	92.06 ± 0.04	40.39 ± 2.32^*∗∗*^
% apoptotic cells	8.40 ± 0.24	7.78 ± 0.6	58.61 ± 2.41^*∗∗*^

Induction of apoptosis was evaluated by flow cytometry of annexin V-PI labeled cells. Mean ± SD of three experiments is shown. ^*∗∗*^ *P* < 0.001 for metformin versus control.

**Table 2:** Cell cycle distribution analysis after metformin treatment.

	Control	4 mM	10 mM
CEM			
G0/G1	63.87 ± 3.68	54.77 ± 2.63^*∗*^	52.98 ± 0.98^*∗*^
S	27.15 ± 1.48	29.44 ± 0.51	32.66 ± 3.53
G2/M	8.97 ± 2.46	15.79 ± 3.11	14.36 ± 3.72
S + G2/M	36.12 ± 3.67	45.23 ± 2.63^*∗*^	47.02 ± 0.98^*∗*^
10E_1_-CEM			
G0/G1	68.55 ± 2.66	61.31 ± 0.95^*∗*^	49.41 ± 5.63^*∗*^
S	24.69 ± 0.76	32.39 ± 0.62	41.18 ± 6.58
G2/M	6.76 ± 2.51	6.29 ± 1.52	9.41 ± 0.95
S + G2/M	31.45 ± 2.66	38.68 ± 0.95^*∗*^	50.59 ± 5.63^*∗*^

Data are shown as cell percentages on different phases of the cell cycle and represent mean ± SD of three experiments. Differences between treatment and control were significant (^*∗*^ *P* < 0.05).

**Table 3:** Densitometric analysis of cell cycle regulatory proteins.

	Control	24 h	48 h	72 h
CEM				
Cdc2 p34	1	0.96 ± 0.19	0.93 ± 0.27	0.47 ± 0.12^*∗*^
Cyclin B1	1	2.37 ± 0.59	0.77 ± 0.36	0.33 ± 0.08^*∗∗*^
Cyclin A	1	3.60 ± 1.25	1.65 ± 0.42	0.73 ± 0.15^NS^
Cyclin E	1	0.84 ± 0.11	1.29 ± 0.08	1.02 ± 0.03^NS^
10E_1_-CEM				
Cdc2 p34	1	1.37 ± 0.32	0.65 ± 0.18	0.12 ± 0.01^*∗∗*^
Cyclin B1	1	1.86 ± 0.85	0.67 ± 0.23	0.15 ± 0.03^*∗∗*^
Cyclin A	1	1.28 ± 0.07	0.61 ± 0.19	0.24 ± 0.04^*∗∗*^
Cyclin E	1	1.12 ± 0.04	0.67 ± 0.13	1.90 ± 0.30^*∗*^
R5-CEM				
Cdc2 p34	1	0.90 ± 0.08	1.31 ± 0.05	2.34 ± 0.91^NS^
Cyclin B1	1	1.45 ± 0.24	2.46 ± 0.59	1.77 ± 0.25^*∗*^
Cyclin A	1	1.35 ± 0.13	1.43 ± 0.36	1.25 ± 0.09^NS^
Cyclin E	1	1.35 ± 0.15	1.47 ± 0.21	1.53 ± 0.16^NS^

Data are expressed as fold changes compared with controls, normalized to actin, and represent mean ± SD of three experiments. ^*∗*^ *P* < 0.05, NS (nonsignificant) for metformin versus control at 72 h.

**Table 4:** Densitometric analysis of PKC*ε* and PKC*δ* levels.

	Control	24 h	48 h	72 h
CEM				
PKC*ε*	1	0.76 ± 0.15	1.23 ± 0.03	0.89 ± 0.06
PKC*δ*	1	1.86 ± 0.58	1.28 ± 0.04	0.65 ± 0.08
10E_1_-CEM				
PKC*ε*	1	0.96 ± 0.16	0.78 ± 0.18	0.87 ± 0.19
PKC*δ*	1	1.05 ± 0.66	0.53 ± 0.34	0.23 ± 0.08
R5-CEM				
PKC*ε*	1	—	0.84 ± 0.35	0.36 ± 0.24
PKC*δ*	1	—	1.13 ± 0.01	2.08 ± 0.20

Data are expressed as fold changes compared with controls, normalized to actin, and represent mean ± SD of three experiments.
